# Association between RBC Indices, Anemia, and Obesity-Related Diseases Affected by Body Mass Index in Iranian Kurdish Population: Results from a Cohort Study in Western Iran

**DOI:** 10.1155/2021/9965728

**Published:** 2021-09-04

**Authors:** Maryam Kohsari, Mehdi Moradinazar, Zohreh Rahimi, Farid Najafi, Yahya Pasdar, Atefeh Moradi, Ebrahim Shakiba

**Affiliations:** Behavioral Disease Research Center, Kermanshah University of Medical Sciences, Kermanshah, Iran

## Abstract

**Objective:**

The relationship between RBC indices and metabolic diseases remains unclear. The association between anemia and obesity is also controversial. The present study aimed to investigate the relationship between RBC indices and metabolic diseases caused by obesity and evaluate the effect of body mass index (BMI) on RBC indices on the Ravansar cohort data.

**Method:**

For the purpose of this study, 9826 participants aged 35–65 years (5158 females and 4668 males) were recruited in the analyses. A quadratic prediction fit plot investigated the association between RBC indices with BMI and lipid profile. The odds ratio of obesity-related diseases in each quartile category of RBC indices and anemia was estimated using multivariable logistic regression models.

**Results:**

Subjects in the fourth quartiles of RBC count, hematocrit (HCT), hemoglobin (HGB), and red cell distribution width (RDW) had a higher risk for obesity-related diseases compared to the first quartiles. However, individuals with the mean corpuscular volume (MCV), mean corpuscular hemoglobin (MCH), and mean corpuscular hemoglobin concentration (MCHC) in fourth quartiles had lower ORs of obesity-related diseases. While BMI reduced the effect of RBC count, HCT, HGB, and RDW on the incidence risk of obesity-related disease, it increased the impact of MCV, MCH, and MCHC. There was a negative association between BMI and RBC indices except for RDW. The BMI effect on RBC indices was different in normal and obese individuals. BMI in mild anemia lowered the risk of metabolic diseases, but it increased the risk of metabolic diseases for moderate anemia.

**Conclusion:**

A higher risk of obesity-related diseases was observed in the fourth quartiles of RBC count, HCT, HGB, and RDW compared to the first quartiles. However, the incidence risk was lower for MCV, MCH, and MCHC. BMI plays an anemia-type dependent role in the relationship. Consideration should be given to the type of anemia in the relationship between BMI and anemia.

## 1. Introduction

Obesity is defined as the body mass index (BMI) ≥30 kg/m^2^ [1]. The rate of obesity has grown so dramatically in the last three decades that in 2014, almost 30% of the world's population was considered overweight and obese, and the number is estimated to reach 50% mark by 2030 [2]. The role of obesity in metabolic diseases including diabetes mellitus (DM), cardiovascular disease (CVD), metabolic syndrome (MetS) [3], and hypertension (HTN) [4] is clearly understood. Obesity has a potent correlation with dyslipidemia that contributes to CVD risk developments [5], and an increase in BMI leads to the progression of heart damage [4] and nonalcoholic fatty liver disease (NAFLD) [6].

Recently, the role of red blood cell (RBC) indices was identified in metabolic diseases. The complete blood count (CBC) test which is routinely administered in medical examinations can be utilized in the early detection of metabolic disorders [7]. However, there are limited studies that indicate the role of RBC indices in the incidence of metabolic diseases. A number of reports have suggested that red cell distribution width (RDW) reduced the risk of MetS [8] and increased the risk of CVD [9] and NAFLD [10]. RDW is an indicator that shows the variation in the size of RBC [9, 10]. NAFLD and CVD are diseases in which inflammation plays an influential role. According to these studies, RDW is associated with inflammation that may increase in response to proinflammatory cytokines. Cytokines can also interact with erythropoietin in the bone marrow, resulting in the lower production of RBC. Besides, cytokines act as RBC suppressors and raise the number of immature RBC, and RDW increased consequently [9, 10].

On the other hand, increased hematocrit (HCT), hemoglobin (HGB), and red blood cell (RBC) count are associated with an increased chance of MetS [11]. Also, it has been suggested that RBC count has a positive relationship with the severity of HTN [12]. This effect may occur as a result of an additional load on the cardiovascular system by increasing RBC count [13].

Various studies indicated a controversial and contradictory relationship between RBC indices and anemia with lipid profile and BMI. Anemia is considered a risk factor for dyslipidemia [14] and CVD [15]. Some studies indicate a lack of association between increased BMI and obesity with anemia [16–18]. A study conducted in China found that the rate of anemia in overweight women was lower compared to normal subjects [18]. Contradictory results were also found on the Iranian population. In a study of young females in north Iran, Rad et al. demonstrated the absence of a significant difference in anemia prevalence between normal weight and obese females [16]. However, a study of young university students in central Iran (males and females) demonstrated a high incidence of anemia among the population with abnormal BMI [19]. Besides, reports indicated the absence of significant correlation [20] and the presence of a negative inverse correlation [21] between mean corpuscular volume (MCV) and BMI. Antwi-Baffour et al. illustrated that the lipid profile parameter is positively associated with RBC count and negatively correlated with HGB and HCT [22]. However, studies have discussed the relationship between inflammation and anemia [23]. We know that obesity is associated with low-grade chronic systemic inflammation. Also, obese people are prone to chronic inflammatory diseases such as DM, MetS, liver, and kidney failure, especially with age [24]. Inflammation in these people eventually leads to the activation of oxidative stress signaling pathways. Free radicals could cause the peroxidation of erythrocyte membrane lipids and activate Ca^**2+**^ permeable nonselective cation channels in the cell membrane. Consequently, phosphatidylserine (PS) translocation enhances from the interior to the cell membrane surface and leads to the erythrocyte suicidal death or eryptosis [25, 26]. In addition, the effect of lipid profile on RBC indices still is ill-defined, although in vitro studies demonstrated that erythrocytes act as a storage of cholesterol for serum lipoproteins, and dyslipidemia may play a role in impairing erythrocyte maturation and deformability [27]. Given that obesity, dyslipidemia, and abnormalities in RBC indices such as anemia all are the risks of CVD. It is crucial to expand our knowledge of the underlying relationships between these factors. As the matter is not also investigated on the Iranian population, the present study is primarily conducted to evaluate the association between RBC indices and obesity-related diseases on a Kurdish population in western Iran. We also aimed to examine the effect of lipid profile parameters and BMI on this relationship.

## 2. Methods

### 2.1. Study Design and Population

The present study used the data obtained from the Ravansar noncommunicable cohort disease (RaNCD) initial phase, which began in 2014 and ended in 2017. The RaNCD cohort study is part of the Iranian adult (PERSIAN) cohort that studies participants in the age range of 35–65 years and aims to conduct a series of follow-ups for a period of 15 years. Study details have been published [28, 29], and all questionnaires, study instructions, and additional information are available at http://persiancohort.com. The study was approved by the Ethics Committees of Kermanshah University of Medical Sciences (KUMS.REC.1394.315), Kermanshah, Iran. Subjects aged 35–65 years who were residents of Ravansar for the past nine months were included in the study after they were fully informed of the process and signed written consent. Individuals with underlying kidney disease (101) and pregnant women (125) were excluded from the study to eliminate confounder variables.

### 2.2. Measurements and Definition

Fasting blood samples were collected by Venoject tubes. After centrifugation for 10 minutes at 300*g*, the samples were transferred to cryotubes and were kept at −20°C until the testing time. Serum triglyceride (TG), high-density lipoprotein cholesterol (HDL-C), total cholesterol (TC), low-density lipoprotein cholesterol (LDL), and fasting blood glucose (FBG) were analyzed with the enzymatic colorimetric assay by the Mindray-BS-380 autoanalyzer (Mindray, USA). RBC indices including RBC count, HCT, HGB, mean corpuscular hemoglobin (MCH), MCHC, and RDW were measured via the CBC test by the Sysmex cell counter. Dyslipidemia was defined based on the presence of one or more abnormalities in the lipid profile, including serum levels of TC ≥ 240 mg/dl, low-density lipoprotein (LDL) 160 mg/dl, triglyceride (TG) ≥150 mg/dl, and high-density lipoprotein (HDL) <40 mg/dl [30]. Blood pressure was measured according to the Joint National Committee on Prevention, Detection, Evaluation, and Treatment of High Blood Pressure (JNC-7) classification of hypertension to diagnose hypertension [31]. After 10 minutes rest, blood pressure was measured twice for each participant using the cuff on both arms at the heart level with one-minute interval between each measurement. The mean obtained for each arm was used as the final blood pressure. Nonalcoholic fatty liver (NAFLD) and cardiovascular diseases (CVD) are based on self-report of participants and use of related medication. NAFLD is reported in nonalcoholic participants with the fatty liver. HTN was defined as SBP ≥140 mm Hg and/or DBP ≥90 m Hg and/or current use of antihypertensive drugs. The presence of three or more of the following criteria identified the existence of MetS: FBS ≥ 100 mg/dl, TG ≥ 150 mg/dl, and reduced HDL-C: <40 mg/dl in males and <50 mg/dl in females, waist circumference (WC) ≥85 cm in males and ≥80 cm in females, and SBP ≥ 130 and DBP ≥ 86 mmHg [32]. Diabetes mellitus was defined as FBS ≥ 126 mg/dl and/or a history of taking medications to treat diabetes [33]. Mild anemia was defined as HGB = 11–11.9 g/dL for females and HGB = 11–12.9 g/dL for males, with moderate anemia as HGB = 8–10.9 g/dL for males and females [34]. The bioimpedance analyzer (BIA) (In Body 770 BIOSPACE, Korea) was used to measure weight. Height was measured with 0.1 cm accuracy using a stadiometer. BMI was calculated by dividing weight (kg) by square of height (m^2^). BMI was categorized into 18.5–24.9 for normal weight, 25–29.9 for overweight, and greater than 30 for obese. An elastic tape was used to measure upper hip bones for waist circumference. The smoking status was specified the National Health Interview Survey (NHIS) [35]. The 24-hour physical activity was determined based on average weekday sport, work, and leisure-related activities, classifying the subjects into three categories of low, moderate, and high physical activities [36].

### 2.3. Statistical Analysis

Quantitative and qualitative variables were analyzed by the *t*-test and chi-square test, respectively. Quadratic prediction fit plot with confidence interval was used to assess the correlation between RBC indices with lipid profile and BMI. The relationship between anemia and the risk of obesity-related disorders was presented within the forest plot with an odds ratio (OR) and 95% confidence interval. The association between RBC indices quartiles with dyslipidemia, HTN, NAFLD, MetS, DM, and CVD was investigated by multivariable logistic regression models. For all tests, the statistical significance was considered at *p* level <0.05. Statistical analyses were carried out using Microsoft Excel 2016 and Stata software (version14.2) (Stata Corp, College Station, TX, USA).

## 3. Results

As given in Table 1, the sample included 5158 females (52.5%) with a mean age of 47.5 ± 8.4 years and 4668 males (47.5%) with a mean age 47.0 ± 8.0 years. Overall, 38% of the subjects were considered healthy, 23.8% had one disorder, and 19.6% and 18.6% suffered from two disorders and more than two obesity-related disorders, respectively.

The prevalence of obesity-related diseases increased with age. Nearly 50% of the individuals over 55 had at least one type of dyslipidemia disorder, and CVD and HTN prevalence doubled compared to the age group of 45–55 years. Subjects with metabolic disorders had higher levels of anthropometric indices and SBP and DBP than the control group. No difference in hypertension parameters was found for NAFLD patients. Participants with obesity-related diseases had higher mean levels of FBG, TC, TG, and LDL-C, but a lower level of HDL-C.

Concerning RBC indices, those who suffered from metabolic diseases had significantly higher RDW and lower MCV and MCH levels. RBC count, HCT, and HGB levels were significantly higher for dyslipidemia subjects. MetS, and DM, with NAFLD and CVD subjects, showed lower levels. There was no difference in RBC count, HCT, HGB, and MCHC between HTN and controls. All participants with metabolic disorders had significantly higher white blood cell (WBC) count. Lymphocyte (lymph) was higher for dyslipidemia and NAFLD patients and lower for HTN and CVD subjects. While only NAFLD showed no difference in monocyte (mono), this was proved to be higher for other obesity-related subjects. Granulocyte percent (GR %) was higher for dyslipidemia, HTN, and CVD subjects, but lower for NAFLD. Platelet (PLT) count was higher in NAFLD, MetS, and DM patients.

**Figure 1:**
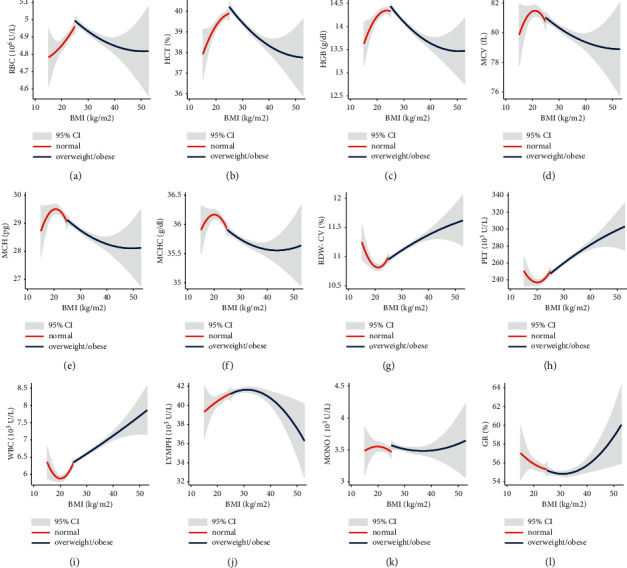
Quadratic fit plots with confidence intervals between BMI and hematological parameters among normal and overweight/obese groups.

**Figure 2:**
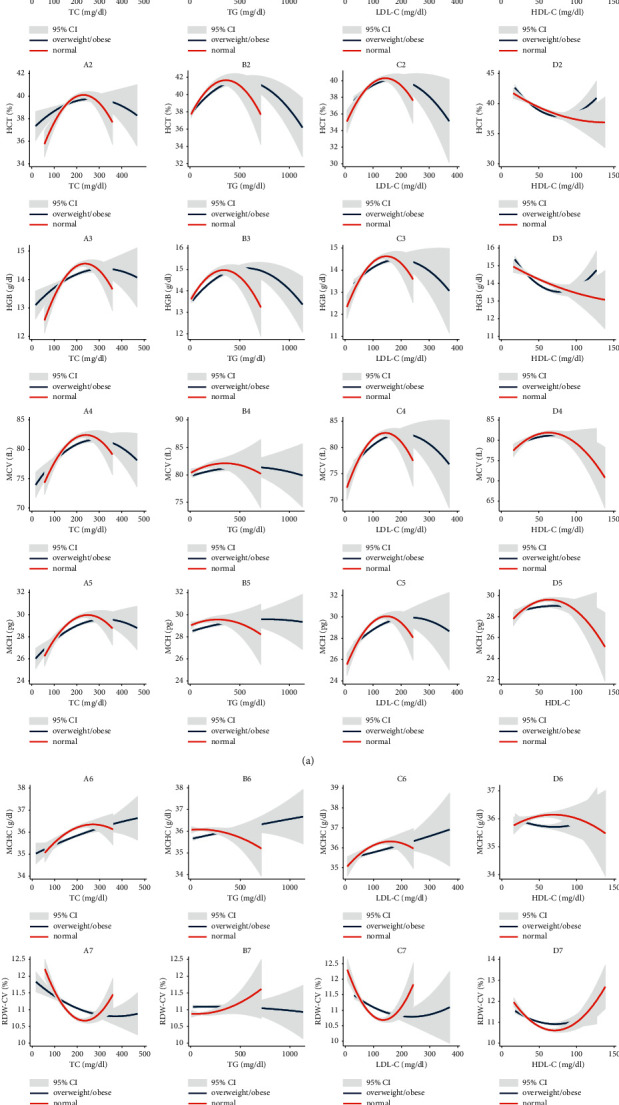
Quadratic fit plots with confidence intervals of the relationship between hematological parameters and lipid profiles parameters among normal and overweight/obese groups.

The correlation between the level of BMI and hematological parameters was examined by quadratic prediction fit plot along with a confidence interval, Figures 1(a)–1(l). The relationship between BMI and RBC indices (including RBC count, HCT, HGB, MCV, MCH, and MCHC) was positive for normal weights and negative for overweight/obese. RDW in normal weights was negatively related to BMI. In the overweight/obese group, the relationship was positive for RDW. Concerning the association between BMI and PLT count, WBC, and GR%, results were similar to the negative correlation in the normal weights versus the positive correlation in the overweight/obese group. BMI and lymphocyte count correlation was positive for normal weight and was negative for overweight/obese. No distinct difference was observed between normal and overweight/obese groups in terms of monocyte count.  Figure 2 shows the relationship between lipid profile parameters and RBC indices. TC and RBC indices (A1–A7) were positively related in the normal range but reversed outside. Regarding the RDW, the correlation was inverse. In the overweight/obese group, this relationship was different. The positive correlation between TC and RBC indices maintained out of the normal range. Besides, in the overweight/obese group outside the normal range, the TC level increases resulted in an increase in RBC count. TG and RBC indices (B1–B7) in the normal and overweight obese groups were almost similar. Except for the MCHC, which was negatively correlated with TG for normal weights and positively correlated with TG for overweight/obese, the correlation pattern between LDL-C and RBC indices (C1–C7) in both normal and overweight/obese groups was the same as TC results. However, the correlation between LDL-C and RBC count was negative for overweight/obese. HDL-C was inversely related to RBC indices (D1–D7). In overweight/obese individuals, the HDL-C had a positive correlation with MCV and MCHC.

Table 2 provides the results of OR with 95% CI according to RBC indices quartiles. After adjusting model 1 for age, gender, smoking status, and physical activity, RBC count, HCT, HGB, and RDW in fourth quartiles had a higher risk for HTN, dyslipidemia, NAFLD, MetS, DM, and CVD compared to the first quartiles. HGB and RBC count in the normal values had the highest risk for NAFLD. Also, there was a similar result of HGB for HTN. On the other hand, the risk of obesity-related disorders decreased within increased MCV, MCH, and MCHC levels. Adjusted model 2 for model 1 plus BMI indicated the dual effects of BMI on the relationship between RBC indices and obesity-related diseases. BMI had a reducing effect on the increased ORs of obesity-related disorders by RBC count, HCT, HGB, and RDW and decreased the risk of these diseases by RBC indices. However, BMI increased the influence of MCV, MCH, and MCHC on obesity-related diseases.

**Figure 3:**
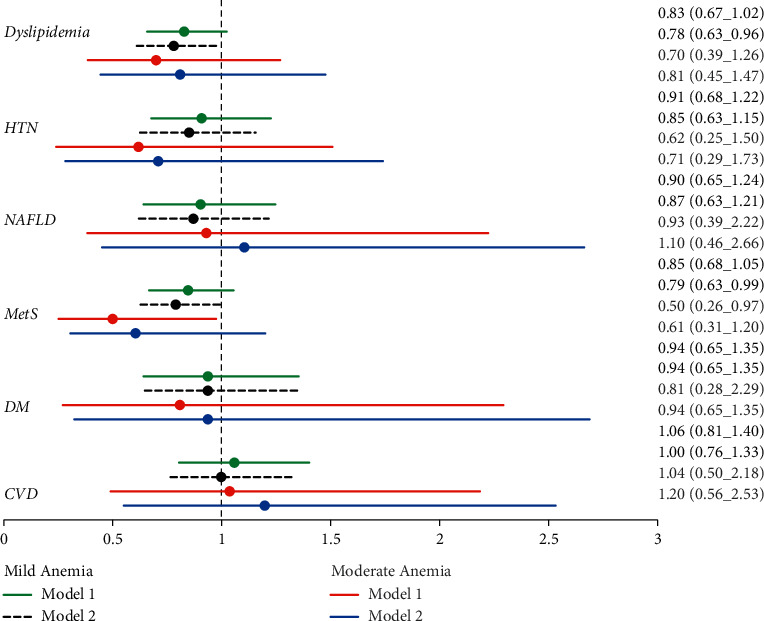
Forest plot of ORs (95% CIs) of obesity-related diseases according to anemia severity.

The association between anemia and the OR of obesity-related diseases is illustrated in Figure 3. In adjusted model 1 for age, gender, smoking status, and physical activity, subjects with mild anemia had 0.83, 0.91, 0.90, 0.85, 0.94, and 1.06-fold risk of dyslipidemia, HTN, NAFLD, MetS, DM, and CVD, respectively, compared with the subject in first quartiles. While the BMI effect in model 2 (model 1 plus BMI) decreased the OR for mild anemia, it increased the risk of obesity-related diseases for the moderate anemia participants.

## 4. Discussion

The current study indicated that the prevalence of dyslipidemia, MetS, CVD, HTN, NAFLD, and DM was 44.2, 33.3, 16.7, 15.5, 10.3, and 8.6% among participants from the Ravansar cohort. According to the literature, no studies have targeted a large homogeneous population in terms of examining the association between complete RBC indices and the risk of metabolic diseases, the effect of BMI alteration on RBC indices, and the influence of lipid profile on hematological indices. Yet we found an increase of WBC, monocytes, PLT counts, and RDW for metabolic diseases. Overweight/obese individuals with increased BMI had also higher WBC, PLT, GR%, and RDW.

Increased WBC count in overweight/obese people can be explained by production of the IL-6, a proinflammatory cytokine in adipose tissue that plays a role in bone marrow granulopoiesis, WBC proliferation, and differentiation [37]. Besides, obesity is associated with impaired glucose tolerance, leading to inflammation in the body tissues [37]. Also, higher levels of WBC, monocyte, and PLT counts in subjects with metabolic diseases, positive association between BMI with WBC, PLT, and monocyte counts, and also GR% in overweight/obese individuals indicate the presence of inflammation in these subjects [38]. Increased thrombocytosis in both individuals with metabolic diseases and those who were overweight/obese could result from an inflammatory process and the activation of platelet which has a key role in atherothrombosis acceleration [39].

In overweight/obese individuals with increasing LDL-C, the levels of RBC count, HGB, HCT, MCV, MCH, MCHC, and RDW decreased. However, decreasing HDL-C was associated with elevation of the RBC count, HGB, HCT, MCV, MCH, and MCHC levels. Increased BMI in overweight/obese subjects was associated with decreased RBC count, HGB, HCT, MCV, MCH, MCHC levels, and lymphocyte counts.

A recent study demonstrated no correlation between BMI and RBC indices except for MCH and MCHC, but it did not specify the relationship and point to a discrepancy by MCH and MCHC between different BMI categories [20]. Alrubaie et al. reported a negative correlation between BMI with MCH and MCV [21]. We noticed a different association between BMI and RBC indices for normal weights against the overweight/obese. This difference can result from proinflammatory cytokines driven from adipocytes and free radicals from oxidative stress. Increased free radicals by affecting RBCs membrane proteins change their natural structure, increase fragility, decrease survival, and cause anisocytosis by the raised proportion of circulating premature erythrocytes [39]. To compensate for the reduction in red blood cell life, the body increases the production of new red blood cells, which has led to an increase in RBC count [27].

In metabolic diseases, we detected increased levels of RBC, HCT, HGB, RDW, and TC. Increased levels of RBC count, HGB, HCT, and RDW were associated with the risk of metabolic diseases. However, enhanced levels of MCV, MCH, and MCHC were associated with reduced risk of metabolic diseases.

In two studies, similar results were obtained, and a positive correlation was detected between HGB, HCT, and RBC count with MetS components, without examination of other RBC indices [7, 11]. Also, Hu et al. demonstrated RDW was a potential prognostic index for liver disease [40]. Furthermore, Jiang et al. determined that HGB can help predict NAFLD [41]. The mechanisms underlying the increased RDW in liver disease are well not understood; however, nutrition deficiency is prevalent in liver disease patients, and reports indicated lower folic acid levels in these patients than healthy controls. Decreased folic acid might affect hematopoiesis and amplify the heterogeneity of RBC [42]. Also, an increase in blood concentration and viscosity causes reduced blood flow rate and blood glucose supply to the muscle, leading to insulin resistance. Insulin resistance is one of the known factors involved in NAFLD pathogenesis that leads to mitochondrion oxidation overload and aggravating fat deposition in liver cells [41, 43]. However, Nebeck et al. reported no differences in RBC indices between MetS individuals and the healthy group [7]. Unlike our findings, Yan et al. reported that an increase in the RDW level was associated with reduced MetS incidence among Chinese population [8]. As the Chinese study was performed on people over 60 years, this contrast might be due to age differences between the two studies. Some evidence suggests that RDW is associated with pulmonary hypertension mortality [40], and RBC count is related to the severity of hypertension [12]. We found no significant difference in RBC count, HTC, HGB, and MCHC levels between HTN subjects and control groups. However, individuals with HTN had higher RDW levels than control subjects. Furthermore, upper levels of RDW, RBC count, HCT, and HGB was correlated with greater risk of HTN. An association between RBC count and HTN may occur due to an additional load on the cardiovascular system by increasing the RBC count [13].

In the present study, moderate anemia was associated with increased BMI and metabolic diseases. Anemia is an independent risk factor for CVD progression and predicts heart complications [44]. It was observed that anemia was the highest risk factor for CVD. RDW is used in the prognosis of CVD and heart failure, and increased RDW could be an important predictor of the mortality and morbidity in atherosclerosis and heart failure, regardless of the level of hemoglobin [43].

We found that BMI decreased the metabolic disease incidence risk in mild anemia and increased it in moderate anemia, indicating that the type and the severity of anemia should be considered when examining the relationship between anemia and obesity. The effect of BMI on the association between obesity-related disorders and RBC indices (including RBC count, HCT, HGB, and RDW) decreased in the ORs. In contrast, BMI increased the incidence risk of metabolic diseases by affecting MCV, MCH, and MCHC, which shows an inverse relationship between BMI and RBC indices based on our findings.

There is presently no consensus in the literature regarding the relationship between anemia and obesity. A study on Chinese women reported a lower prevalence of anemia in overweight females compared to normal weight participants [18]. Findings of two studies on Iranian population suggested no significant difference in the prevalence of anemia and the levels of hemoglobin, MCV, serum iron, ferritin, and transferrin according to BMI [16, 45]. In contrast, evidence exists that indicate an association between obesity and anemia [19, 46]. The role of iron deficiency in obesity is unclear. However, obesity, as a low-grade inflammation status, may cause a negative regulation of iron absorption through increased secretion of hepcidin by adipocytes and result in a decrease in iron uptake in small intestine [45, 46]. Current results demonstrated that HGB and HDL-C levels were negatively correlated with obesity. The association between HDL-C and MCV was also a positive linear correlation in overweight/obese. Studies have shown that HDL-C levels are inversely related to the incidence of anemia. HDL-C is positively associated with MCV, which is likely to play a role in megaloblastic anemia [27]. Although dyslipidemia, MetS, and DM group had higher mean of RBC count, their level of MCV and MCH was lower compared to the controls.

Being the first study of an Iranian population on a large scale, our findings imply that obesity affected the lipid profile and influenced the RBC indices. Decreased HGB and increased RDW levels with consequent anemia and the elevation of PLT, WBC count, and reduction of lymphocytes resulted in inflammation with BMI playing an important role in the process. Abnormal lipid profile in overweight/obese had an inverse relation with RBC indices. We observed an inflammatory state with increased WBC, monocytes, and PLT counts and also changes in all RBC indices for metabolic diseases. Changes in RBC indices in overweight/obese had a significant impact on the interpretation of laboratory results. Finally, it should be noted that the current study is an explorative study aiming to create new hypotheses which should be further investigated in future studies.

## 5. Conclusion

We found an increase of WBC, monocytes, PLT counts, and RDW for metabolic diseases. There was also a correlation between increased levels of RBC count, HGB, HCT, and RDW and the risk of metabolic diseases. Increased BMI enhanced the WBC, PLT, GR counts, and RDW for overweight/obese. An inverse correlation between LDL-C and the levels of RBC count, HGB, HCT, MCV, MCH, MCHC, and RDW was also observed for them. Furthermore, their increased BMI was associated with reduced RBC count, HGB, HCT, MCV, MCH, MCHC levels, and lymphocyte counts. While the risk of obesity-related diseases in the fourth quartiles of RBC count, HCT, HGB, and RDW was higher than the first quartiles; MCV, MCH, and MCHC had lower OR. Moderate anemia was associated with increased BMI and metabolic diseases. Further studies on inflammation signaling pathways in adipose tissue are needed to achieve accurate results.

## Figures and Tables

**Table 1:** General and biochemical characteristics of participants.

Variables	Dyslipidemia		HTN		NAFLD		MetS		DM		CVD	
	Yes	No	Yes	No	Yes	No	Yes	No	Yes	No	Yes	No
Number %	4344 (44.2)	5482 (55.8)	1527 (15.5)	8298 (84.5)	1008 (10.3)	8818 (89.7)	3432 (33.3)	6393 (66.7)	846 (8.6)	8932 (91.4)	1639 (16.7)	8187 (83.3)
Males	2475 (53)	2193 (47)^c^	673 (14.4)	3995 (85.6)	306 (6.7)	4362 (93.4)^c^	1605 (34.4)	3063 (65.6)	379 (8.2)	4262 (91.8)	567 (12.2)	4101 (87.8)
Age group												
35–45	1901 (40.9)	2751 (59.1)^c^	292 (6.3)	4360 (93.7)^c^	418 (9)	4234 (91)^c^	1266 (27.2)	3386 (72.8)^c^	198 (4.3)	4425 (95.7)^c^	310 (6.7)	4342 (93.3)^c^
46–55	1444 (46)	1692 (54)	554 (17.7)	2581 (82.3)	380 (12.1)	2756 (87.9)	1247 (39.8)	1888 (60.2)	343 (11)	2776 (89)	561 (17.9)	2575 (82.1)
56–65	999 (49)	1039 (51)	681 (33.4)	1357 (66.6)	210 (10.3)	1828 (89.7)	919 (45.1)	1119 (54.9)	305 (15)	1731 (85)	768 (37.7)	1270 (62.3)
Smoke	519 (45.1)	631 (54.9)^b^	186 (16.2)	964 (83.8)^c^	117 (10.2)	1033 (89.8)^c^	399 (34.7)	751 (65.3)^c^	101 (8.8)	1044 (91.2)^c^	190 (16.5)	960 (83.5)^c^
Physical activity daily METs												
Low (24–36.5)	1446 (49.3)	1489 (50.7)^c^	525 (17.9)	2410 (82.1)^c^	365 (12.4)	2570 (87.6)^c^	1184 (40.3)	1751 (59.7)^c^	300 (10.3)	2618 (89.7)^c^	594 (20.2)	2341 (79.8)^c^
Moderate (36.6–44.9)	2014 (41.8)	2803 (58.2)	731 (15.2)	4086 (84.8)	519 (10.8)	4298 (89.2)	1691 (35.1)	3126 (64.9)	417 (8.7)	4379 (91.3)	808 (16.8)	4009 (83.2)
High (≥45)	884 (42.6)	1190 (57.4)	271 (13)	1802 (87)	124 (6)	1950 (94)	557 (26.9)	1516 (73.1)	129 (6.2)	1935 (93.8)	237 (11.4)	1837 (88.6)
BMI												
Normal	926 (21.3)	1910 (34.8)^c^	320 (20.9)	2516 (30.3)^c^	120 (11.9)	2716 (30.8)^c^	487 (14.1)	2349 (36.7)^c^	148 (17.5)	2680 (30.0)^c^	320 (19.5)	2516 (30.7)^c^
Overweight	2108 (48.5)	2126 (38.7)	641 (41.9)	3592 (43.2)	448 (44.4)	3786 (89.4)	1676 (48.8)	2557 (39.9)	360 (42.5)	3846 (43.0)	688 (41.9)	3546 (43.3)
Obesity	1310 (30.2)	1446 (26.5)	566 (37.2)	2190 (26.5)	440 (43.7)	2316 (84)	1269 (37.1)	1487 (23.4)	338 (40)	2406 (26.9)	631 (38.6)	2125 (26.0)

Mean ± SD												
WC (cm)	98.7 ± 9.6	96.2 ± 11.0^c^	100.1 ± 10.5	96.8 ± 10.4^c^	102.1 ± 9.9	96.7 ± 10.4^c^	100.8 ± 9.1	95.4 ± 10.7^c^	100.9 ± 9.8	96.9 ± 10.5^c^	100.5 ± 10.5	96.6 ± 10.4^c^
SBP (mmHg)	110.4 ± 17.3	106.3 ± 16.5^c^	129.0 ± 21.9	104.3 ± 12.5^c^	108.6 ± 16.2	108.1 ± 17.1	114.5 ± 19.3	104.8 ± 14.5^c^	115.3 ± 18.0	107.5 ± 16.7^c^	119.5 ± 20.3	105.8 ± 15.2^c^
DBP (mmHg)	71.0 ± 10.1	68.8 ± 9.6^c^	80.5 ± 12.2	67.8 ± 7.9^c^	69.8 ± 9.6	69.8 ± 9.9	73.0 ± 11.1	68.0 ± 8.7^c^	72.8 ± 10.6	69.5 ± 9.8^c^	74.9 ± 11.2	68.7 ± 9.3^c^
RBC (10^6^ *μ*/L)	5.01 ± 0.5	4.8 ± 0.5^c^	4.9 ± 0.5	4.9 ± 0.5	4.8 ± 0.01	4.9 ± 0.006^b^	4.9 ± 0.5	4.8 ± 0.5^c^	4.99 ± 0.5	4.91 ± 0.5^b^	4.8 ± 0.5	4.9 ± 0.5^c^
Hct (%)	40.1 ± 4.1	38.9 ± 4.0^c^	39.4 ± 4.2	39.4 ± 4.1	38.8 ± 3.9	39.5 ± 4.1^c^	39.7 ± 4.1	39.3 ± 4.1^c^	39.7 ± 3.9	39.4 ± 4.1^a^	38.8 ± 4.1	39.6 ± 4.1^c^
MCV (fL)	80.4 ± 6.9	80.8 ± 7.0^b^	80.2 ± 7.2	80.7 ± 6.9^a^	80.2 ± 6.8	80.6 ± 7.0	80.3 ± 6.9	80.7 ± 7.0^b^	80.0 ± 6.3	80.7 ± 7.0^b^	80.3 ± 7.0	80.7 ± 7.0^a^
HGB (g/L)	14.4 ± 1.5	13.9 ± 1.5^c^	14.1 ± 1.6	14.1 ± 1.5	13.8 ± 1.5	14.2 ± 1.5^c^	14.2 ± 1.5	14.1 ± 1.5^c^	14.2 ± 1.5	14.1 ± 1.5	13.8 ± 1.5	14.2 ± 1.5^c^
MCH (pg)	28.9 ± 3.0	28.9 ± 3.0	28.7 ± 3.0	28.9 ± 3.0^b^	28.6 ± 3.0	28.9 ± 3.0^b^	28.8 ± 3.0	29.0 ± 3.0^b^	28.6 ± 2.8	28.9 ± 3.0^b^	28.7 ± 3.0	28.9 ± 3.0^b^
MCHC (g/dl)	35.9 ± 1.4	35.8 ± 1.5^b^	35.8 ± 1.4	35.8 ± 1.5	35.6 ± 1.6	35.8 ± 1.4^c^	35.8 ± 1.5	35.8 ± 1.4	35.7 ± 1.7	35.8 ± 1.4	35.7 ± 1.4	35.8 ± 1.5^a^
RDW-CV (%)	11.1 ± 0.9	10.9 ± 1.0^c^	11.09 ± 0.9	11.01 ± 0.9^b^	11.2 ± 1.1	11.0 ± 0.9^c^	11.1 ± 0.9	10.9 ± 1.0^c^	11.0 ± 0.8	11.0 ± 0.9^a^	11.1 ± 1.0	11.0 ± 0.9^b^
WBC (10^3^ *μ*/L)	6.6 ± 1.6	6.2 ± 1.5^c^	6.5 ± 1.6	6.4 ± 1.5^b^	6.6 ± 1.5	6.4 ± 1.6^c^	6.7 ± 1.5	6.2 ± 1.5^c^	7.0 ± 1.7	6.3 ± 1.5^c^	6.5 ± 1.6	6.4 ± 1.5^c^
Lymphocyte (10^3^ *μ*/L)	41.5 ± 8.8	40.8 ± 8.8^b^	40.3 ± 8.9	41.3 ± 8.7^c^	41.7 ± 8.7	41.1 ± 8.8^a^	41.3 ± 8.7	41.0 ± 8.8	41.2 ± 9.0	41.1 ± 8.8	40.5 ± 9.0	41.3 ± 8.7^b^
Monocyte (10^3^ *μ*/L)	3.5 ± 1.2	3.4 ± 1.2^c^	3.6 ± 1.3	3.4 ± 1.2^C^	3.5 ± 1.3	3.5 ± 1.2	3.5 ± 1.2	3.4 ± 1.2^a^	3.6 ± 1.3	3.5 ± 1.2^a^	3.5 ± 1.3	3.5 ± 1.2^a^
GR %	54.8 ± 9.4	55.6 ± 9.3^c^	56.0 ± 9.4	55.1 ± 9.3^b^	54.7 ± 9.2	55.3 ± 9.4^a^	55.0 ± 9.3	55.4 ± 9.4	55.1 ± 9.6	55.2 ± 9.3	55.9 ± 9.5	55.1 ± 9.3^b^
PLT (10^3^ *μ*/L)	254.5 ± 60.9	253.0 ± 62.5	254.2 ± 64.8	253.5 ± 61.2	264.4 ± 64.3	252.4 ± 61.4^c^	259.0 ± 61.9	250.8 ± 61.5^c^	259.8 ± 62.7	253.1 ± 61.7^b^	256.2 ± 65.2	253.2 ± 61.1
TC (mg/dl)	190.7 ± 46.7	181.3 ± 28.4^c^	189.6 ± 39.4	184.7 ± 37.6^c^	186.9 ± 37.9	185.3 ± 38.0	191.3 ± 39.1	182.4 ± 37.0^c^	190.2 ± 43.7	185.0 ± 37.3^b^	187.4 ± 40.1	185.1 ± 37.5^a^
TG (mg/dl)	182.0 ± 99.8	102.0 ± 38.0^c^	151.7 ± 91.0	134.9 ± 80.5^c^	150.3 ± 88.4	136.1 ± 81.6^c^	197.7 ± 98.8	105.6 ± 47.9^c^	179.5 ± 119.0	133.6 ± 76.9^c^	150.4 ± 87.6	134.9 ± 81.1^c^
HDL (mg/dl)	39.8 ± 10.1	51.6 ± 9.3^c^	45.9 ± 11.0	46.5 ± 11.3	45.42 ± 11.0	46.5 ± 11.3^b^	40.0 ± 8.7	49.7 ± 11.1^c^	44.0 ± 11.0	46.6 ± 11.3^c^	46.1 ± 11.2	46.4 ± 11.3
LDL (mg/dl)	105.9 ± 30.1	99.0 ± 20.4^c^	104.5 ± 26.2	101.6 ± 25.2^c^	102.5 ± 25.8	102.0 ± 25.4	105.7 ± 26.0	100.1 ± 24.9^c^	104.0 ± 28.1	101.9 ± 25.1^a^	102.9 ± 26.6	101.9 ± 25.2
FBS (mg/dl)	101.0 ± 34.5	93.8 ± 25.6^c^	105.5 ± 39.0	95.4 ± 27.9^c^	102.9 ± 33.7	96.3 ± 29.6^c^	109.7 ± 39.6	90.3 ± 20.7^c^	167.8 ± 62.9	90.3 ± 9.8^c^	107.7 ± 40.2	94.8 ± 27.1^c^

^a^*P* value <0.05. ^b^*P* value <0.01. ^c^*P* value <0.001.

**Table 2:** Odds ratio (ORs) and 95% CI of obesity-related diseases (according to quartiles of RBC indices).

RBC indices			HTN OR (95% CI)	Dyslipidemia OR (95% CI)	NAFLD OR (95% CI)	MetS OR (95% CI)	DM OR (95% CI)	CVD OR (95% CI)
RBC count	**Q1 < 4.53**	Reference	1.00	1.00	1.00	1.00	1.00	1.00
	**Q2 4.54_4.87**	Model 1	1.03 (0.82_1.30)	1.06 (0.90_1.25)	1.02 (0.79_1.31)	**1.24 (1.04_1.47)**	1.26 (0.93_1.70)	0.86 (0.69_1.07)
		Model 2	0.99 (0.79_1.25)	1.01 (0.86_1.20)	0.95 (0.74_1.23)	1.16 (0.97_1.39)	1.20 (0.88_1.63)	0.82 (0.66_1.02)
	**Q3 4.89_5.24**	Model 1	**1.28 (1.01_1.62)**	**1.39 (1.17_1.65)**	1.34 (1.03_1.74)	**1.64 (1.37_1.96)**	**1.84 (1.36_2.50)**	1.11 (0.88_1.39)
		Model 2	1.20 (0.95_1.53)	**1.27 (1.07_1.52)**	1.21 (0.93_1.58)	**1.44 (1.20_1.74)**	**1.68 (1.23_2.28)**	1.03 (0.82_1.30)
	**Q4 > 5.25**	Model 1	**1.56 (1.22_2.00)**	**1.72 (1.43_2.05)**	1.26 (0.94_1.68)	**2.05 (1.70_2.47)**	**2.10 (1.53_2.88)**	1.16 (0.91_1.48)
		Model 2	**1.38 (1.07_1.78)**	**1.52 (1.26_1.82)**	1.08 (0.81_1.46)	**1.73 (1.42_2.11)**	**1.84 (1.33_2.53)**	1.03 (0.81_1.33)

Hct	**Q1 < 36.7**	Reference	1.00	1.00	1.00	1.00	1.00	1.00
	**Q2 36.8_39.4**	Model 1	0.92 (0.73_1.16)	1.07 (0.91_1.26)	1.00 (0.79_1.27)	**1.20 (1.02_1.43)**	1.21 (0.91_1.60)	0.89 (0.72_1.10)
		Model 2	0.94 (0.74_1.18)	1.07 (0.91_1.27)	1.01 (0.79_1.28)	**1.22 (1.02_1.45)**	1.18 (0.89_1.57)	0.88 (0.71_1.10)
	**Q3 39.5_42.2**	Model 1	1.23 (0.97_1.56)	**1.25 (1.05_1.49)**	1.14 (0.88_1.49)	**1.40 (1.17_1.69)**	1.03 (0.75_1.40)	1.05 (0.83_1.32)
		Model 2	1.20 (0.94_1.54)	**1.21 (1.01_1.45**)	1.05 (0.80_1.38)	**1.35 (1.12_1.64)**	0.97 (0.71_1.33)	1.03 (0.81_1.30)
	**Q4 > 42.3**	Model 1	**1.36 (1.03_1.78)**	**1.40 (1.15_1.70)**	1.21 (0.87_1.68)	**1.62 (1.32_2.00)**	1.26 (0.89_1.77)	1.07 (0.81_1.40)
		Model 2	1.27 (0.96_1.68)	**1.29 (1.05_1.58)**	1.10 (0.79_1.54)	**1.47 (1.19_1.83)**	1.12 (0.79_1.59)	1.01 (0.77_1.34)

HGB	**Q1 < 13.2**	Reference	1.00	1.00	1.00	1.00	1.00	1.00
	**Q2 13.3_14.2**	Model 1	0.97 (0.78_1.22)	1.03 (0.88_1.21)	0.88 (0.69_1.12)	1.02 (0.86_1.20)	1.00 (0.75_1.32)	1.13 (0.91_1.40)
		Model 2	1.00 (0.80_1.26)	1.06 (0.90_1.24)	0.88 (0.69_1.12)	1.05 (0.88_1.25)	0.98 (0.74_1.30)	1.16 (0.93_1.44)
	**Q3 14.3_15.2**	Model 1	1.22 (0.96_1.54)	1.16 (0.97_1.37)	1.02 (0.78_1.32)	**1.22 (1.02_1.46)**	1.06 (0.79_1.43)	1.11 (0.88_1.41)
		Model 2	1.25 (0.98_1.58)	1.16 (0.98_1.39)	1.00 (0.76_1.30)	1.25 (1.04_1.50)	1.04 (0.77_1.41)	1.13 (0.89_1.44)
	**Q4 > 15.3**	Model 1	1.20 (0.92_1.57)	**1.47 (1.21_1.78)**	0.94 (0.67_1.30)	**1.47 (1.20_1.79)**	1.18 (0.85_1.66)	1.01 (0.76_1.34)
		Model 2	1.16 (0.88_1.52)	**1.41 (1.16_1.71)**	0.88 (0.63_1.23)	**1.39 (1.13_1.71)**	1.09 (0.78_1.54)	1.00 (0.75_1.32)

MCV	**Q1 < 78**	Reference	1.00	1.00	1.00	1.00	1.00	1.00
	**Q2 78.1_81.6**	Model 1	0.90 (0.72_1.12)	1.23 (1.05_1.45)	1.00 (0.77_1.29)	0.93 (0.79_1.09)	1.09 (0.84_1.42)	1.04 (0.84_1.30)
		Model 2	0.92 (0.74_1.16)	**1.28 (1.09_1.51)**	1.03 (0.80_1.33)	0.98 (0.83_1.16)	1.11 (0.85_1.46)	1.10 (0.88_1.37)
	**Q3 81.7_84.7**	Model 1	**0.80 (0.64_0.99)**	0.97 (0.83_1.14)	0.88 (0.67_1.14)	**0.81 (0.69_0.96)**	0.79 (0.60_1.05)	0.91 (0.73_1.14)
		Model 2	0.83 (0.66_1.04)	1.02 (0.87_1.20)	0.94 (0.72_1.23)	0.88 (0.74_1.05)	0.83 (0.62_1.02)	0.98 (0.78_1.23)
	**Q4 > 84.8**	Model 1	**0.75 (0.60_0.94)**	0.82 (0.69_0.96)	1.01 (0.78_1.30)	**0.74 (0.63_0.88)**	**0.57 (0.42_0.77)**	0.89 (0.71_1.11)
		Model 2	0.82 (0.65_1.03)	0.90 (0.76_1.06)	1.13 (0.87_1.47)	0.72 (0.72_1.02)	**0.63 (0.46_0.85)**	0.97 (0.77_1.21)

MCH	**Q1 < 27.8**	Reference	1.00	1.00	1.00	1.00	1.00	1.00
	**Q2 27.9_29.5**	Model 1	0.98 (0.78_1.21)	**1.19 (1.02_1.40)**	1.09 (0.85_1.39)	0.97 (0.83_1.15)	1.02 (0.78_1.32)	1.03 (0.82_1.28)
		Model 2	0.97 (0.78_1.21)	**1.22 (1.04_1.43)**	1.10 (0.85_1.41)	0.99 (0.83_1.17)	1.01 (0.77_1.32)	1.07 (0.85_1.33)
	**Q3 29.6_30.8**	Model 1	0.81 (0.65_1.02)	0.91 (0.77_1.06)	0.95 (0.74_1.23)	**0.81 (0.69_0.96)**	**0.73 (0.55_0.96)**	0.93 (0.75_1.62)
		Model 2	0.85 (0.68_1.07)	0.94 (0.80_1.11)	1.00 (0.78_1.30)	0.87 (0.74_1.04)	**0.75 (0.56_0.99)**	1.00 (0.80_1.25)
	**Q4 > 30.9**	Model 1	0.77 (0.61_0.96)	0.88 (0.75_1.03)	0.81 (0.62_1.06)	**0.75 (0.64_0.89)**	**0.60 (0.45_0.81)**	0.81 (0.65_1.01)
		Model 2	0.84 (0.66_1.05)	0.98 (0.83_1.15)	0.93 (0.71_1.22)	0.88 (0.74_1.04)	**0.68 (0.50_0.91)**	0.91 (0.72_1.15)

MCHC	**Q1 < 35.2**	Reference	1.00	1.00	1.00	1.00	1.00	1.00
	**Q2 35.3_36.1**	Model 1	1.13 (0.91_1.41)	**1.27 (1.09_1.49)**	1.01 (0.79_1.28)	1.08 (0.92_1.27)	1.02 (0.78_1.34)	1.00 (0.81_1.24)
		Model 2	1.13 (0.90_1.41)	**1.30 (1.11_1.53)**	1.01 (0.79_1.29)	1.08 (0.91_1.28)	1.01 (0.77_1.33)	1.01 (0.82_1.26)
	**Q3 36.2_36.9**	Model 1	1.07 (0.85_1.33)	1.07 (0.91_1.25)	**0.71 (0.55_0.92)**	0.91 (0.77_1.07)	0.93 (0.70_1.22)	0..91 (0.73_1.13)
		Model 2	1.12 (0.89_1.40)	1.13 (0.96_1.32)	**0.74 (0.57_0.95)**	0.98 (0.82_1.16)	0.96 (0.72_1.27)	0.97 (0.78_1.21)
	**Q4 > 37**	Model 1	0.94 (0.74_1.96)	1.15 (0.98_1.36)	**0.68 (0.52_0.90)**	0.81 (0.81_1.14)	0.91 (0.68_1.22)	0.82 (0.65_1.04)
		Model 2	1.03 (0.81_1.31)	**1.26 (1.06_1.48)**	**0.75 (0.57_0.99)**	1.08 (0.91_1.29)	0.98 (0.73_1.31)	0.92 (0.72_1.16)

RDW	**Q1 < 10.4**	Reference	1.00	1.00	1.00	1.00	1.00	1.00
	**Q2 10.5_10.9**	Model 1	1.04 (0.84_1.30)	**1.56 (1.31_1.81)**	1.30 (0.99_1.69)	**1.46 (1.23_1.72)**	1.09 (0.82_1.45)	1.17 (0.94_1.46)
		Model 2	0.97 (0.78_1.22)	**1.45 (1.24_1.69)**	1.16 (0.89_1.53)	**1.31 (1.10_1.59)**	0.98 (0.74_1.31)	1.10 (0.87_1.37)
	**Q3 11_11.4**	Model 1	1.23 (0.98_1.54)	**2.15 (1.83_2.52)**	**1.49 (1.14_1.96)**	**2.06 (1.74_2.44)**	1.26 (0.95_1.68)	**1.31 (1.05_1.65)**
		Model 2	1.06 (0.84_1.34)	**1.93 (1.63_2.27)**	1.28 (0.97_1.68)	**1.73 (1.45_2.06)**	1.09 (0.81_1.45)	1.13 (0.90_1.42)
	**Q4 > 11.4**	Model 1	1.21 (0.96_1.52)	**1.91 (1.62_2.26)**	**1.80 (1.38_2.35)**	**2.06 (1.73_2.45)**	**1.46 (1.10_1.94)**	1.22 (0.97_1.53)
		Model 2	1.04 (0.82_1.31)	**1.66 (1.40_1.97)**	**1.48 (1.12_1.94)**	**1.68 (1.40_2.01)**	1.21 (0.90_1.62)	1.04 (0.82_1.31)

^*∗*^Model 1 adjusted for age, gender, and smoking and physical activity. ^*∗∗*^Model 2 adjusted for model 1 plus BMI.

## Data Availability

The datasets used and/or analyzed during the current study are available from the corresponding author upon reasonable request.

## Additional Points

*Limitations of the Study*. Since the study was cross-sectional, a prospective study is needed to confirm the results. The inflammatory and lipid peroxidation indices were not examined in our population.
